# Antimicrobial susceptibility in *Neisseria gonorrhoeae* and epidemiological data of gonorrhoea patients in five cities across Ethiopia, 2021–22

**DOI:** 10.1093/jacamr/dlae002

**Published:** 2024-02-01

**Authors:** Muluken Birhanu, Woldaregay Erku Abegaz, Daniel Schröder, Adane Mihret, Tamrat Abebe, Susanne Jacobsson, Geremew Tasew, Tesfa Addis, Abera Abdeta, Yonas Alem, Zelealem Desalegn, Muluneh Ademe, Birhanu Teka, Meron Yohannes, Mahlet Yigeremus, Daniel Golparian, Solomon Gebre-Selassie, Magnus Unemo

**Affiliations:** Department of Medical Laboratory Science, College of Health Science, Assosa University, Assosa, Ethiopia; Department of Microbiology, Immunology and Parasitology, School of Medicine, College of Health Sciences, Addis Ababa University, Addis Ababa, Ethiopia; Department of Microbiology, Immunology and Parasitology, School of Medicine, College of Health Sciences, Addis Ababa University, Addis Ababa, Ethiopia; Department of Laboratory Medicine, Microbiology, Faculty of Medicine and Health, WHO Collaborating Centre for Gonorrhoea and Other STIs, Örebro University, Örebro, Sweden; Department of Microbiology, Immunology and Parasitology, School of Medicine, College of Health Sciences, Addis Ababa University, Addis Ababa, Ethiopia; Department of Bacteriology, Armauer Hansen Research Institute, Addis Ababa, Ethiopia; Department of Microbiology, Immunology and Parasitology, School of Medicine, College of Health Sciences, Addis Ababa University, Addis Ababa, Ethiopia; Department of Laboratory Medicine, Microbiology, Faculty of Medicine and Health, WHO Collaborating Centre for Gonorrhoea and Other STIs, Örebro University, Örebro, Sweden; National Clinical Bacteriology and Mycology Reference Laboratory, Ethiopian Public Health Institute, Addis Ababa, Ethiopia; National Clinical Bacteriology and Mycology Reference Laboratory, Ethiopian Public Health Institute, Addis Ababa, Ethiopia; National Clinical Bacteriology and Mycology Reference Laboratory, Ethiopian Public Health Institute, Addis Ababa, Ethiopia; Department of Medical Laboratory Sciences, Ambo University, Ambo, Ethiopia; Department of Microbiology, Immunology and Parasitology, School of Medicine, College of Health Sciences, Addis Ababa University, Addis Ababa, Ethiopia; Department of Microbiology, Immunology and Parasitology, School of Medicine, College of Health Sciences, Addis Ababa University, Addis Ababa, Ethiopia; Department of Microbiology, Immunology and Parasitology, School of Medicine, College of Health Sciences, Addis Ababa University, Addis Ababa, Ethiopia; Department of Medical Laboratory Science, College of Health Sciences, Addis Ababa University, Addis Ababa, Ethiopia; Department of Gynecology and Obstetrics, School of Medicine, College of Health Sciences, Addis Ababa University, Addis Ababa, Ethiopia; Department of Laboratory Medicine, Microbiology, Faculty of Medicine and Health, WHO Collaborating Centre for Gonorrhoea and Other STIs, Örebro University, Örebro, Sweden; Department of Microbiology, Immunology and Parasitology, School of Medicine, College of Health Sciences, Addis Ababa University, Addis Ababa, Ethiopia; Department of Laboratory Medicine, Microbiology, Faculty of Medicine and Health, WHO Collaborating Centre for Gonorrhoea and Other STIs, Örebro University, Örebro, Sweden; Institute for Global Health, University College London (UCL), London, UK

## Abstract

**Introduction:**

Antimicrobial resistance (AMR) in *Neisseria gonorrhoeae* is a global public health concern and enhanced global gonococcal AMR surveillance is imperative. As in many African countries, regular, representative and quality-assured gonococcal AMR is lacking in Ethiopia. We describe the AMR in gonococcal isolates from five cities across Ethiopia, 2021–22, and patient epidemiological data.

**Methods:**

Urethral discharge from males and cervical discharge from females were collected from October 2021 to September 2022. Epidemiological data were collected using a questionnaire. MIC determination (ETEST; eight antimicrobials) was performed on gonococcal isolates and EUCAST breakpoints (v13.1) were used.

**Results:**

From 1142 urogenital swab samples, 299 species-identified gonococcal isolates were identified; 78.3% were from males and 21.7% from females. The median age for males and females was 25 and 23 years, respectively. Most isolates (61.2%) were identified in Addis Ababa, followed by Gondar (11.4%), Adama (10.4%), Bahir Dar (10.0%) and Jimma (7.0%). The resistance level to ciprofloxacin, tetracycline and benzylpenicillin was 97.0%, 97.0% and 87.6%, respectively, and 87.6% of isolates were producing β-lactamase. All isolates were susceptible to ceftriaxone, cefixime, azithromycin and spectinomycin. Recommended therapy [ceftriaxone (250 mg) plus azithromycin (1 g)] was used for 84.2% of patients.

**Conclusions:**

We present the first national quality-assured gonococcal AMR data from Ethiopia. Resistance levels to ciprofloxacin, tetracycline and benzylpenicillin were exceedingly high. However, all isolates were susceptible to ceftriaxone, cefixime, azithromycin and spectinomycin. In Ethiopia, it is essential to strengthen the gonococcal AMR surveillance by including further epidemiological data, more isolates from different cities, and WGS.

## Introduction

Increasing prevalence of gonorrhoea and antimicrobial resistance (AMR) in *Neisseria gonorrhoeae* are global public health concerns and the extended-spectrum cephalosporins (ESCs) ceftriaxone and cefixime are the only remaining options for first-line empirical antimicrobial monotherapy of gonorrhoea.^1,2^ However, gonococcal strains with resistance to ceftriaxone and/or cefixime have been reported globally.^2–5^ Enhanced, regular and quality-assured gonococcal AMR surveillance is imperative to inform national and international treatment guidelines, public health policy and action.^2,6^ Nevertheless, in most African countries, having the highest gonorrhoea incidence, the surveillance of aetiologically diagnosed gonorrhoea and gonococcal AMR is exceedingly limited, lacking representativeness and epidemiological data, or completely absent.^2,6–8^

In Ethiopia, since 2001 STI management has been based on a syndromic approach.^9–13^ Consequently, aetiological gonorrhoea diagnostics including gonococcal culture and AMR testing has been nearly lacking during recent decades. A gonococcal AMR survey in 2013–14 identified high levels of ciprofloxacin resistance, which resulted in revisions of the treatment guidelines, i.e. ciprofloxacin was replaced with ceftriaxone (250 mg) combined with azithromycin (1 g) for first-line empirical gonorrhoea therapy.^13^ Some minor previous Ethiopian studies have reported high levels of ESC resistance, i.e. 48.0% resistance, 35.8% resistance and 27.8% non-susceptibility to ceftriaxone in Gondar,^11^ Debre Markos^14^ and Bahir Dar,^9^ respectively. Furthermore, 37.5% resistance and 15% non-susceptibility to cefixime were reported in Bahir Dar^10^ and Addis Ababa,^15^ respectively. However, these studies mostly examined low numbers of presumptive gonococcal isolates (no gonococcal-confirmatory assay used), lacked appropriate quality assurance and epidemiological data of patients, and AMR was determined using the disc diffusion method, which is suboptimal for *N. gonorrhoeae*.

This study aimed to (i) investigate the antimicrobial susceptibility of gonococcal isolates from patients in five cities across Ethiopia, 2021–22; and (ii) assess epidemiological data of the gonorrhoea patients.

## Materials and methods

### Study sites, biological samples and epidemiological data

Twenty-seven health facilities were included in the following five different cities across Ethiopia: capital city Addis Ababa (Central Ethiopia), Adama (Southeast Ethiopia), Gondar (Northwest Ethiopia), Bahir Dar (Northwest Ethiopia) and Jimma (Southwest Ethiopia). Consecutive males with urethral discharge and females with vaginal discharge aged ≥15 years from October 2021 to September 2022 were invited to participate. After written informed consent was obtained, two urethral specimens from males and two endocervical specimens from females were collected using Dacron swabs and placed in Amies transport medium with charcoal for transportation to the laboratory within 1–3 h. Patients with complicated STIs, other serious illnesses, suspected sexual abuse, and menstruating females were excluded. Epidemiological data were collected using a questionnaire, which was translated into local languages. After sample collection, all STI patients were treated according to the National STI Management Guidelines of Ethiopia.^13^

### Microbiological culture for N. gonorrhoeae

One urethral or endocervical swab was used for Gram-stained microscopy and the other for culture on modified Thayer–Martin (MTM) agar plates incubated at 35°C ± 1°C in a humid 3%–5% CO_2_-enriched atmosphere for up to 72 h. Suspected gonococcal colonies were species confirmed based on colony morphology, identification of Gram-negative diplococci in microscopy, rapid oxidase positivity, superoxol test and MALDI-TOF MS (Bruker Daltonics, GmbH, Germany).

### Antimicrobial susceptibility testing of N. gonorrhoeae

The MICs (mg/L) of eight antimicrobials were determined using the ETEST (bioMérieux, Marcy-l’Étoile, France) on GC agar plates, as described.^16^ The 2016 WHO gonococcal strains were used for quality control.^16^ Clinical breakpoints recommended by EUCAST (v13.1) were used, where available.^17^ For azithromycin and gentamicin, the EUCAST epidemiological cut-off (ECOFF)^17^ and published gentamicin breakpoints,^18^ respectively, were used. β-Lactamase production was detected by chromogenic cephalosporin test.

### Ethics

The study was approved by the Department Research Ethics review committee (MF/MICRO/135/2012) from the College of Health Science Institutional Review Board of Addis Ababa University (CHS/RTD/279/2020, CHS/RTD/30/2022) and from the National Research and Ethical Review Committee (02/256/882/14).

## Results

### N. gonorrhoeae isolates

Among 1172 study participants, 1142 were eligible and gave consent to participate. Of the urogenital swab samples (*n* = 1142), 39.4% were collected in Addis Ababa and 13.3%–17.0% in the other four cities. In total, 299 (26.2%) *N. gonorrhoeae* isolates from 297 patients were identified. Most of the isolates (61.2%) were identified in Addis Ababa (Table 1).

**Table 1.** The geographic distribution of *N. gonorrhoeae* isolates identified in five cities across Ethiopia from October 2021 to September 2022

City	Region of Ethiopia	Number (%) of urogenital swabs cultured	Number (%) of confirmed *N. gonorrhoeae* isolates
Addis Ababa	Central	450 (39.4)	183 (61.2)
Adama	Southeast	194 (17.0)	31 (10.4)
Gondar	Northwest	182 (15.9)	34 (11.4)
Bahir Dar	Northwest	164 (14.4)	30 (10.0)
Jimma	Southwest	155 (13.3)	21 (7.0)
Total		1142 (100)	299 (100)

### Epidemiological data of gonorrhoea patients

The epidemiological data of the gonorrhoea patients have been summarized in Table S1 (available as Supplementary data at *JAC-AMR* Online). Briefly, most gonococcal isolates were cultured from males (78.3%) and the median ages for the males and females were 25 and 23 years, respectively. Most of the patients were treated with the recommended first-line ceftriaxone 250 mg plus azithromycin 1 g therapy (84.2%, *n* = 250). However, 7.0% (*n* = 21) were treated with second-line spectinomycin 2 g plus azithromycin 1 g, 5.4% (*n* = 16) with ceftriaxone 250 mg plus doxycycline, and 3.4% (*n* = 10) with ceftriaxone 250 mg plus azithromycin 1 g plus metronidazole.

### Antimicrobial susceptibility and resistance

All isolates were susceptible to ceftriaxone, cefixime, azithromycin and spectinomycin. However, 97.0%, 97.0% and 87.6% were resistant to ciprofloxacin, tetracycline and benzylpenicillin, respectively, and 26.8% were intermediate susceptible to gentamicin (Table S2).^18^ Furthermore, 87.6% were producing β-lactamase (Table S2). Accordingly, all resistance to benzylpenicillin was β-lactamase plasmid-mediated (no chromosomally mediated penicillin resistance was detected). Notably, three (1.1%) β-lactamase-producing isolates were not benzylpenicillin resistant based on the MIC (1 mg/L), i.e. only bordering resistance according to the EUCAST resistance breakpoint (MIC > 1 mg/L),^17^ but reported as benzylpenicillin resistant as all β-lactamase-producing gonococcal isolates should be reported.^17^

The MIC distribution for ceftriaxone, cefixime, azithromycin and spectinomycin ranged from ≤0.002–0.032, ≤0.016–0.125, ≤0.016–1 and 8–32 mg/L, respectively (Figure S1). Almost all (98%) isolates had low ceftriaxone MICs (≤0.016 mg/L). One isolate (0.3%) had a cefixime MIC of 0.125 mg/L, i.e. at the EUCAST clinical resistance breakpoint (resistance, MIC > 0.125 mg/L).^17^ Furthermore, 1 isolate (0.3%) had an azithromycin MIC of 1 mg/L, i.e. at the EUCAST azithromycin ECOFF (resistance, MIC > 1 mg/L),^17^ and 19 isolates (6.4%) had an azithromycin MIC of 0.5 mg/L (Figure S1).

## Discussion

This is the first national study describing the *N. gonorrhoeae* antimicrobial susceptibility in five cities across Ethiopia during 2021–22 using MIC determination (ETEST), which was quality assured in accordance with WHO standards and controls.^2,6,16^ Furthermore, epidemiological data from the gonorrhoea patients were analysed.

All isolates were susceptible to ceftriaxone and cefixime, which is promising because ceftriaxone (250 mg) plus azithromycin (1 g) is the recommended first-line empirical treatment for gonorrhoea in Ethiopia.^13^ Almost all isolates (98%) had low ceftriaxone MIC values (≤0.016 mg/L) and only six isolates (2.0%) had the highest ceftriaxone MIC (0.032 mg/L). Similarly, 95% of the isolates had low cefixime MICs (≤0.016 mg/L). Our findings were consistent with some minor studies from Ethiopia, which reported 100% ceftriaxone susceptibility.^10,12,15^ However, in several Ethiopian studies, high levels of resistance^11,14^ or non-susceptibility^9^ to ceftriaxone and high-level resistance^10^ or non-susceptibility^15^ to cefixime have been reported. Notably, in these studies, the examined isolates were few and frequently not species-confirmed as *N. gonorrhoeae*, antimicrobial susceptibility testing was performed using only the disc diffusion method, and the antimicrobial susceptibility testing was not sufficiently quality assured. Our findings of high susceptibility to ESCs were in agreement with studies from other African countries such as Uganda, South Africa, Kenya, Cameroon, Cote d’Ivoire, Zimbabwe and Ghana.^8^ Nevertheless, resistance to both ceftriaxone and cefixime have been reported from many other countries worldwide.^2–6^ Consequently, it cannot be excluded that gonococcal strains with ESC resistance will be imported, and the vast misuse of antimicrobials by healthcare providers, unskilled practitioners and drug consumers in Ethiopia,^19^ and many other African countries, may also select for ESC-resistant strains. Therefore, enhanced, regular and quality-assured surveillance of gonococcal AMR is imperative.

Azithromycin is a component of the dual antimicrobial therapy recommended for empirical treatment of uncomplicated gonorrhoea. However, data on the azithromycin susceptibility in Ethiopia have been lacking. All gonococcal isolates in the present study were susceptible to azithromycin, and 93.3% of the isolates had low azithromycin MIC values (≤0.25 mg/L). Our results in Ethiopia were in line with studies in several other African countries such as South Africa, Kenya, Uganda and Guinea-Bissau.^8^ However, the resistance to azithromycin has rapidly increased in many countries globally.^2–6^

Spectinomycin (2 g) is the second-line therapy for the treatment of uncomplicated gonorrhoea in Ethiopia. All of the gonococcal isolates were susceptible to spectinomycin, which is in concordance with global findings.^5,20^ In contrast, previous Ethiopian studies have reported a high prevalence of spectinomycin resistance^10^ or intermediate resistance.^12^ However, these studies have only used disc diffusion methods for antimicrobial susceptibility testing and additionally suffered from insufficient quality assurance.

The limitations of this study included that no samples from extragenital sites (anorectum and oropharynx) were collected and that few of the enrolled patients reported regarding sexual orientation or sexual practices.

In conclusion, we present the first national quality-assured gonococcal AMR data from Ethiopia. Resistance levels to ciprofloxacin, tetracycline and benzylpenicillin were exceedingly high. All isolates were susceptible to ceftriaxone, cefixime, azithromycin and spectinomycin; however, occasional isolates bordering resistance to both cefixime and azithromycin were found. In Ethiopia, it is imperative to enhance the aetiological diagnosis of gonorrhoea and strengthen the gonococcal AMR surveillance by including additional epidemiological data, anorectal and oropharyngeal samples, more isolates from representative geographical locations, and ideally WGS.

## Supplementary Material

dlae002_Supplementary_Data

## Supplementary data

Tables S1 and S2, and Figure S1 are available as Supplementary data at *JAC-AMR* Online.
