# Supplementary material for: Antimicrobial susceptibility in Neisseria gonorrhoeae and epidemiological data of gonorrhoea patients in five cities across Ethiopia, 2021–22
Source: JAC Antimicrob Resist. 2024 Feb 1;6(1):dlae002. doi: 10.1093/jacamr/dlae002 (PMC10833647; doi:10.1093/jacamr/dlae002)
Supplement: dlae002_Supplementary_Data [file dlae002_supplementary_data.zip › JAC-AMR-2023-183_Supplementary Tables S1 and S2.pdf]

Table S1. Epidemiological data of gonorrhoea patients in five cities across Ethiopia from October 2021 to September 2022

Variable	Category	Number (%) of patients with gonorrhoea
Age (years)	15-19	27 (9.1)
	20-24	122 (41.1)
	25-34	131 (44.1)
	35-44	17 (5.7)
Sex	Male	232 (78.1)
	Female	65 (21.9)
Marital status	Unmarried (single)	206 (69.4)
	Married	76 (25.6)
	Divorced	15 (5.0)
Sex worker	Yes	37 (12.5)
	No	260 (87.5)
Long distance drivers	Yes	11 (3.7)
	No	286 (96.3)
Multiple sexual partner	Yes	187 (63.0)
	No	110 (37.0)
Unprotected sexual intercourse	Vaginal	24 (8.1)
	Oral	0 (0)
	Vaginal and oral	8 (2.7)
	Unknown	265 (89.2)
Therapy	CRO 250 mg + AZM 1 g	250 (84.2)
	CRO 250 mg + DOX	16 (5.4)
	SPT 2 g + AZM 1 g	21 (7.0)
	CRO 250 mg + AZM 1 g + MTZ	10 (3.4)
Took antibiotics prior to visiting the health facility?	Yes	55 (18.5)
	No	242 (81.5)
From where was antibiotic obtained?	Drug vendors	50 (16.8)
	Private clinic	4 (1.3)
	Public Health Centre or Hospital	1 (0.03)

	NA	242 (81.5)
Know the name of drug taken	Yes	20 (67.3)
	No	35 (32.7)
Name of the antibiotic	CIP	13 (65.0)
	NOR	5 (25.0)
	CRO	2 (10.0)

AZM, azithromycin; CIP, ciprofloxacin; CRO, ceftriaxone; DOX, doxycycline; NA, not applicable; NOR, norfloxacin; SPT, spectinomycin

Table S2. Susceptibility to four antimicrobials[a] and β-lactamase production of *Neisseria gonorrhoeae* isolates (n=299) collected across Ethiopia from October 2021 to September 2022

**Antimicrobial**	**Susceptibility**	**City**					
		**Addis Ababa** (n=183, 61.2%)	**Adama** (n=31, 10.4%)	**Gondar** (n=34, 11.1%)	**Bahir Dar** (n=30, 10.1%)	**Jimma** (n=21, 7.1%)	**Total** (n=299, 100%)
Ciprofloxacin	Susceptible (MIC ≤0.03 mg/L)[17]	3 (1.6%)	2 (6.4%)	3 (8.8%)	0 (0%)	1 (4.8%)	9 (3.0%)
	Resistant (MIC >0.06 mg/L)[17]	180 (98.4%)	29 (93.6%)	31 (91.2%)	30 (100%)	20 (95.2%)	290 (97.0%)
Gentamicin	Susceptible (MIC ≤4 mg/L)[18]	128 (69.9%)	26 (83.9%)	23 (67.6%)	28 (93.3%)	14 (66.7%)	219 (73.2%)
	Intermediate susceptible (MIC: 8-16 mg/L)[18]	55 (30.1%)	5 (16.1%)	11 (32.4%)	2 (6.7%)	7 (33.3%)	80 (26.8%)
	Resistant (MIC >16 mg/L)[18]	0 (0%)	0 (0%)	0 (0%)	0 (0%)	0 (0%)	0 (0%)
Tetracycline	Susceptible (MIC ≤0.5 mg/L)[17]	5 (3.8%)	4 (12.9%)	0 (0%)	0 (0%)	0 (0%)	9 (3.7%)
	Resistant (MIC >0.5 mg/L)[17]	178 (96.2%)	27 (87.1%)	34 (100%)	30 (100%)	21 (100%)	290 (97.0%)

Benzylpenicillin	Susceptible (MIC ≤0.06 mg/L)[17]	27 (14.8%)	5 (16.1%)	3 (8.8%)	4 (13.3%)	1 (4.8%)	40 (13.4%)
	Resistant (MIC >1 mg/L)[17]	156 (85.2%)	26 (83.9%)	31 (91.2%)	26 (86.7%)	20 (95.2%)	259[b] (86.6%)
β-lactamase	Positive	158 (86.3%)	27 (87.1%)	31 (91.2%)	26 (86.7%)	20 (95.2%)	262[b] (87.6%)
	Negative	25 (13.2%)	4 (12.9%)	3 (8.8%)	4 (13.3%)	1 (4.8%)	37 (12.4%)

[a]All isolates were susceptible to ceftriaxone, cefixime, azithromycin and spectinomycin.

[b]Three (1.1%) β-lactamase-producing isolates were not benzylpenicillin resistant based on the MIC (1 mg/L), i.e. only bordering resistance according to the EUCAST resistance breakpoint (MIC>1 mg/L),[17] but reported as benzylpenicillin resistant as all β-lactamase-producing gonococcal isolates should be reported.[17]
