# Supplementary material for: Antimicrobial susceptibility in Neisseria gonorrhoeae and epidemiological data of gonorrhoea patients in five cities across Ethiopia, 2021–22
Source: JAC Antimicrob Resist. 2024 Feb 1;6(1):dlae002. doi: 10.1093/jacamr/dlae002 (PMC10833647; doi:10.1093/jacamr/dlae002)
Supplement: dlae002_Supplementary_Data [file dlae002_supplementary_data.zip › JAC-AMR-2023-183_Supplementary figure S1.pdf]

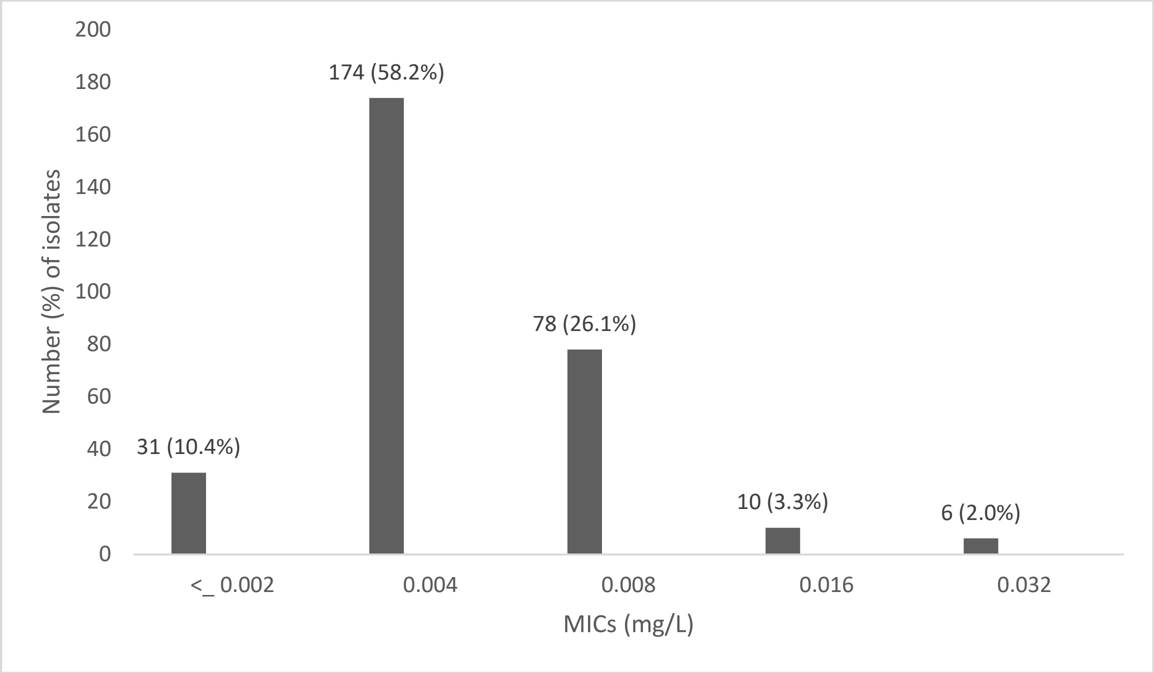

200
180
160
140
120
100
80
60
40
20
0
Number (%) of isolates
174 (58.2%)
78 (26.1%)
31 (10.4%)
10 (3.3%)
6 (2.0%)
<_ 0.002
0.004
0.008
0.016
0.032
MICs (mg/L)

A.

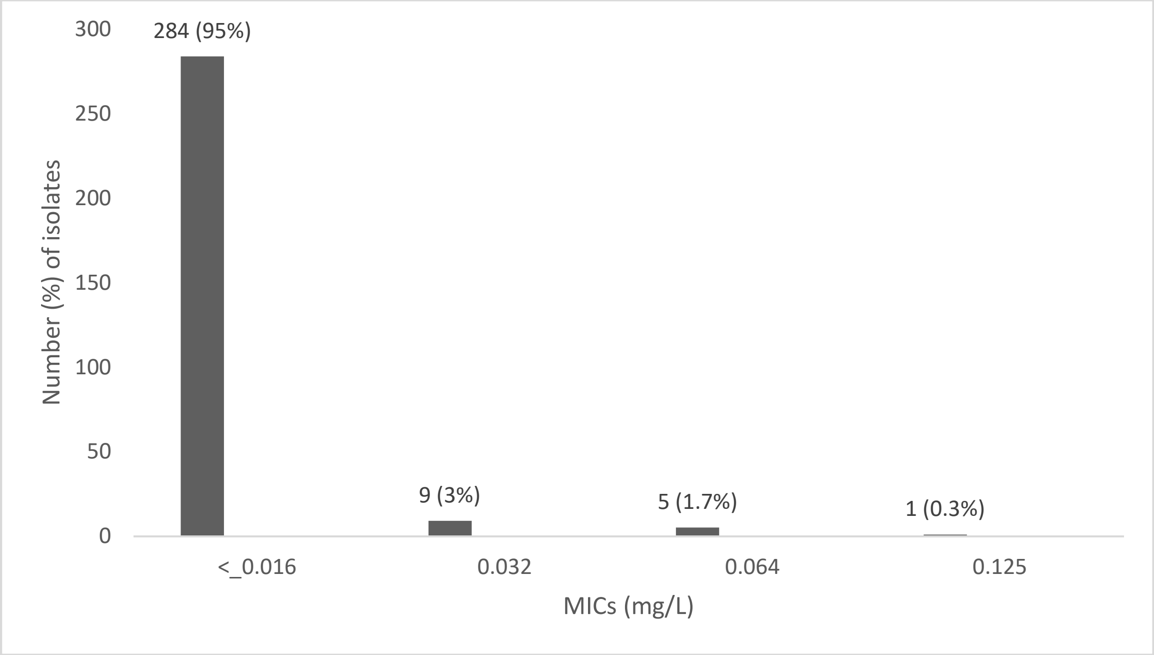

300
250
200
150
100
50
0
Number (%) of isolates
284 (95%)
9 (3%)
5 (1.7%)
1 (0.3%)
<_0.016
0.032
0.064
0.125
MICs (mg/L)

B.

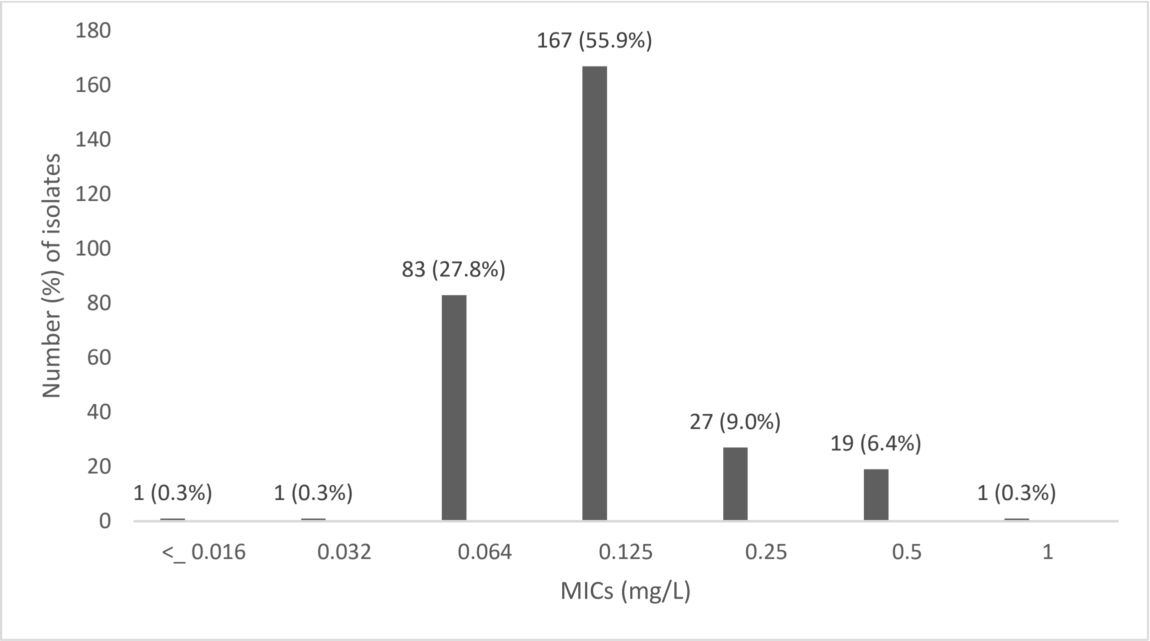

C.

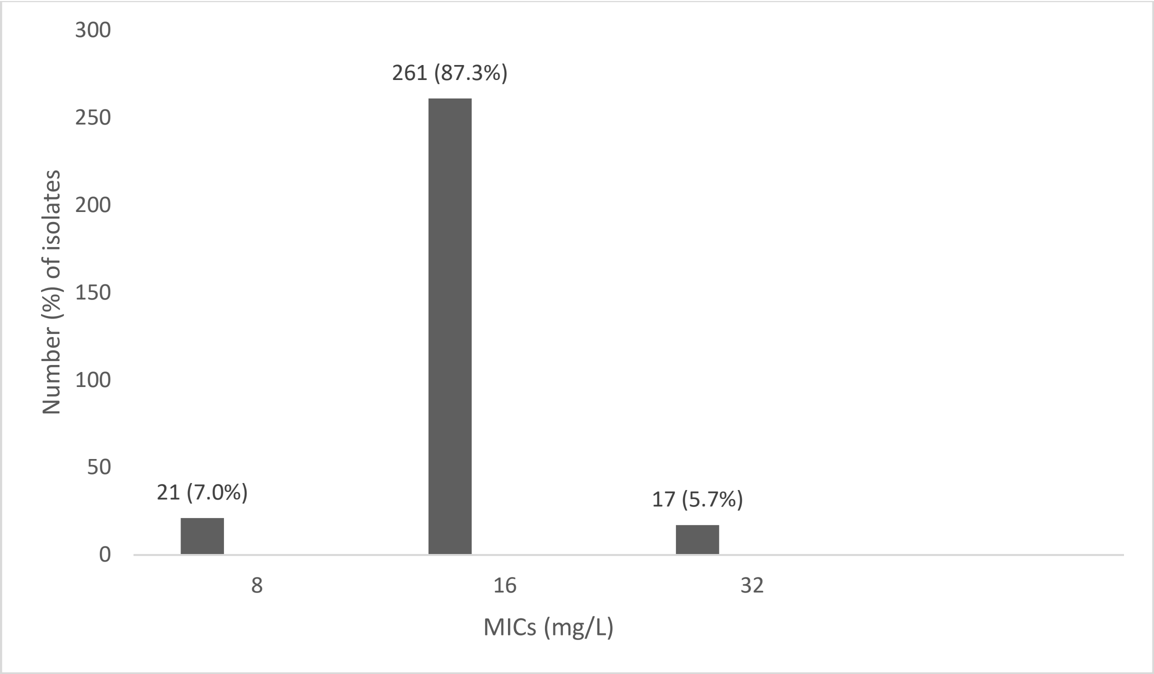

D.

Figure S1. MIC values (mg/L) for *Neisseria gonorrhoeae* isolates (n=299) for A. ceftriaxone, B. cefixime, C. azithromycin, and D. spectinomycin identified in five cities across Ethiopia from October, 2021 to September, 2022. Only whole MIC doubling dilutions are reported.
